# Back to Basics: Pitting Edema and the Optimization of Hypertension Treatment in Incident Peritoneal Dialysis Patients (BRAZPD)

**DOI:** 10.1371/journal.pone.0036758

**Published:** 2012-05-23

**Authors:** Sebastião R. Ferreira-Filho, Gilberto R. Machado, Valéria C. Ferreira, Carlos F. M. A. Rodrigues, Thyago Proença de Moraes, José C. Divino-Filho, Marcia Olandoski, Christopher McIntyre, Roberto Pecoits-Filho, on behalf of the BRAZPD study investigators

**Affiliations:** 1 Nefroclínica de Uberlândia, Minas Gerais, Brazil; 2 Federal University of Uberlândia, Minas Gerais, Brazil; 3 Center for Health and Biological Sciences, Pontifícia Universidade Católica do Paraná, Curitiba, Brazil; 4 Baxter Healthcare, Division of Baxter Novum and Renal Medicine, CLINTEC, Karolinska Institute, Stockholm, Sweden; 5 Faculty of Medicine & Health Sciences, University of Nottingham, Nottingham, United Kingdom,; University of Sao Paulo Medical School, Brazil

## Abstract

Systemic arterial hypertension is an important risk factor for cardiovascular disease that is frequently observed in populations with declining renal function. Initiation of renal replacement therapy at least partially decreases signs of fluid overload; however, high blood pressure levels persist in the majority of patients after dialysis initiation. Hypervolemia due to water retention predisposes peritoneal dialysis (PD) patients to hypertension and can clinically manifest in several forms, including peripheral edema. The approaches to detect edema, which include methods such as bioimpedance, inferior vena cava diameter and biomarkers, are not always available to physicians worldwide. For clinical examinations, the presence of pitting located in the lower extremities and/or over the sacrum to diagnose the presence of peripheral edema in their patients are frequently utulized. We evaluated the impact of edema on the control of blood pressure of incident PD patients during the first year of dialysis treatment. Patients were recruited from 114 Brazilian dialysis centers that were participating in the BRAZPD study for a total of 1089 incident patients. Peripheral edema was diagnosed by the presence of pitting after finger pressure was applied to the edematous area. Patients were divided into 2 groups: those with and without edema according to the monthly medical evaluation. Blood arterial pressure, body mass index, the number of antihypertensive drugs and comorbidities were analyzed. We observed an initial BP reduction in the first five months and a stabilization of blood pressure levels from five to twelve months. The edematous group exhibited higher blood pressure levels than the group without edema during the follow-up. The results strongly indicate that the presence of a simple and easily detectable clinical sign of peripheral edema is a very relevant tool that could be used to re-evaluate not only the patient's clinical hypertensive status but also the PD prescription and patient compliance.

## Introduction

Cardiovascular disease is the most common cause of morbidity and mortality in patients with chronic kidney disease (CKD) [1]–[3]. Systemic arterial hypertension (SAH) is an important risk factor for cardiovascular disease and is frequently observed in this population along with a decline of renal function [4]. Although overload and renal replacement therapy (RRT) with dialysis usually improve fluid balance and partially remove uremic toxins, high blood pressure levels may persist after the initiation of dialysis, and hypertension is present in the majority of both peritoneal and hemodialysis patients [5], [6].

The reduction in blood pressure levels observed in peritoneal dialysis (PD) patients can be attributed to the continuous effective control of fluid balance and, consequently, extracellular volume [7]; however, this reduction is not always sustained. In fact, higher than normal blood pressure levels are observed in many patients during dialysis therapy, mainly due to the limitations in achieving normal fluid status [8]–[10]. Hypervolemia due to water retention predisposes PD patients to hypertension [11], [12] and can manifest clinically in several forms, including peripheral edema [9]. Detecting occult edema often involves the measurement of metrics such as bioimpedance, inferior vena cava diameter and biomarkers, but these methods are not available to all physicians. To detect edema in their patients, many doctors have at their disposal only the presence of pitting located in the lower extremities and/or over the sacrum.

Despite the fact that some patients present SAH independently of volemic status, it is recognized that hypervolemia, with or without the presence of edema, is one of the principal factors responsible for the resistance of PD patients to SAH treatment [13], [14]. Blood pressure normalization often requires modifications to the ultrafiltration target, an increase in sodium removal, a decrease in fluid and sodium intake, blood sugar control and/or an increase in the number of prescribed hypertension drugs [6], [7], [15], [21]. Considering that the expansion of extracellular volume can occur during dialysis and that peripheral edema detectable on a physical exam can be the result of a hypervolemic state [13], little is known about the correlations between pitting edema and blood pressure control in hypertensive patients receiving PD treatment.

We hypothesized that the presence of pitting edema is associated with the worsening of SAH, which leads to the cardiovascular impact observed in fluid-overloaded patients. Thus, in the present study, we evaluated the impact of peripheral edema on hypertensive control in incident PD patients with SAH during the first year of dialysis treatment.

## Methods

Each consecutive incident patient recruited from 114 Brazilian dialysis centers participating in the BRAZPD study from December 2004 through October 2007 was included, totaling 3439 patients. Incident patients were defined as patients who originated from pre-dialysis conservative treatment or HD, who started treatment with PD during the study period and who remained on the therapy for at least 90 days. In Brazil, 60% of the patients start treatment in APD and 40% in CAPD. Details of the BRAZPD study design and characteristics of the cohort are described elsewhere [16]. Briefly, after being selected to participate in the study, each clinic submitted the project to its local ethics committee (the protocol was approved by the ethics committees of Federal University of Uberlandia), and all patients signed an informed consent. Physician and nurses at each dialysis center were trained by the study monitors to use the clinical research software *PDnet*, which was designed specifically to collect data for this study. From a total of 3439 incident patients, 239 were excluded because they were less than 18 years old, 1650 were excluded for not completing 12 full months of follow up (i.e., patients who missed at least one medical evaluation monthly for 12 consecutive months, or who dropped out due to hemodialysis, transplant or death), 430 were excluded because they were normotensive with or without previously using any antihypertensive drugs and because they did not have peripheral edema at the beginning of the PD treatment, and 31 were excluded due to missing data. After exclusion criteria were applied, 1089 hypertensive patients were included in the analysis.

The variables analyzed included anthropomorphic data, comorbidities, systolic arterial pressure (SAP), diastolic arterial pressure (DAP), mean arterial pressure (MAP), erythropoietin use, PD modality (CAPD or APD), and physical examination. During the physical examination, peripheral edema was characterized by the presence of pitting after finger pressure was applied to the edematous area for at least five seconds. The nephrologists graded pitting edema on a scale from 1+ to 4+. The urea and plasma creatinine, serum potassium, and hemoglobin values of the patients were measured to be used as annual means.

For all patients, the dialysis nurse or the nephrologist measured blood pressure during their monthly visits to the dialysis clinic. For the diagnosis of systemic hypertension, the following WHO/ISH criteria were applied: SAP≥140 mmHg and/or DAP≥90 mmHg, with or without the use of hypertensive medication. SAP levels were verified using an oscillating method. Mean arterial pressure was calculated using the formula MAP = (2DAP+SAP)/3. The number of anti-hypertensive drug classes used monthly by the patients (NAC) was also reported. The classes considered were diuretics, beta-blockers, ACE inhibitors, angiotensin II receptor blockers, centrally and peripherally acting alpha-blockers, and calcium channel blockers. Each class listed was counted as one unit, and the NAC represented the mathematical mean of the number of anti-hypertensive drug classes used per patient for each subgroup.

**Figure 1:**
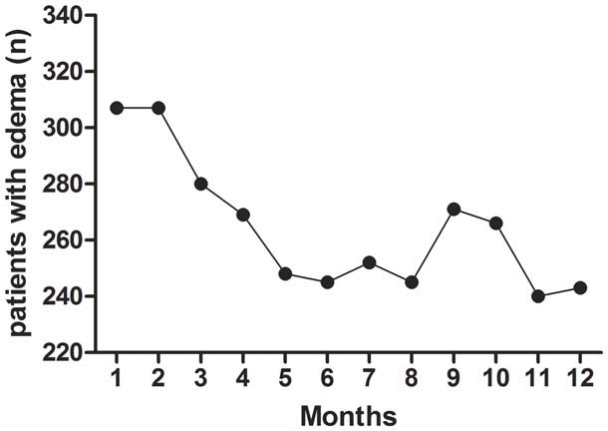
Number of patients/month with clinically detectible edema.

After the exclusion criteria were applied, the final sample consisted of 1089 hypertensive patients. These patients were subdivided into those with (E+) and without (E−) clinically detectable pitting edema, according to the monthly medical evaluation at both the beginning of the observation period and during the twelve months of follow up. The number of patients in each subgroup varied monthly depending on the presentation of edema at that particular evaluation (Figure 1). In order to analyze the trend for edema and high blood pressure levels, we also monitored for 12 months the patients classified E+ and E− based on the first month classification.

### Statistical Analysis

Categorical variables are presented as frequencies and percentages. Continuous variables are presented as the mean ± standard deviation (mean ± SD). In the figures, continuous variables are presented as the mean ± standard error. The chi-squared test and analysis of variance (ANOVA), with repeat measures and measures of position and distribution, were utilized for the comparison between the E+ and E− subgroups. The parallelism analysis of both groups was performed to verify the trends and similarities between the groups, for the initial defined groups at month 1. For all analyses, a p-value of <0.05 was considered statistically significant. All statistical analyses were performed using SPSS version 8.0 (Chicago, IL, USA).

## Results

Descriptive data at baseline PD treatment level (after the first month on PD) for all patients included in this study are shown in Table 1. The mean patient age was 58.2±15.3 years, and more than half (56.9%) of the patients were female. The mean SBP was 156.7±18.7 mmHg, the mean DBP was 90.0±12.7 mmHg, and the mean MAP was 112.2±12.8 mmHg. The mean body mass index (BMI) was 25.4±5.0 kg/m^2^. The correlation between BMI and the number of patients with edema was negative and significant (r = −0.83). The increase of blood pressure (SBP, DBP and MAP) correlated with the number of patients with edema: 0.76; 0.69 and 0.52 respectively (p<0.001).Overall, 42.6% of study participants were diabetic, and the mean number of anti-hypertensive class drugs (NAC) used was 2.1±1.0 drugs/patient. Forty-three percent of patients were on APD using Homechoice™ (Baxter Healthcare) as the cycler, and all patients were prescribed only glucose-based PD solutions (Dianeal, Baxter Healthcare).

**Table 1:** Demographic, clinical and laboratory characteristics of patients at the baseline evaluation.

Variable	Total	Patients		
	population	with edema (E+)	without edema (E−)	*P* value
Number of patients (n)	1089	307	782	<0.001
Age (year)	58.2±15.3	59. 6±14.3	57.7±15.6*	0.03
Female (%)	56.9	55.7	57.4	0.61
Diabetes (%)	42.6	56.0	37.3*	<0.0001
Race (%)				
Asian	2.7	3.2	2.8	0.92
White	61.7	61.6	61.1	0.96
Black	35.6	35.2	36.1	0.93
Height (cm)	161.6±10.0	161.6±10.5	161.7±9.8	0.44
Weight (Kg)	66.7±15.0	69.8±14.5	65.5±15.1*	<0.0001
Body mass index (Kg/m2)	25.4±5.0	26.7±5.1	24.9±4.9*	<0.0001
SAP (mmHg)	156.7±18.7	159.5±19.6	155.6±18.2*	0.001
DAP (mmHg)	90.0±12.7	90.7±13.3	89.7±12.5	0.11
MAP (mmHg)	112.2±12.8	113.7±13.4	111.7±12.6*	0.01
NCA	2.1±1.0	2.3±1.0	2.0±0.7*	<0.0001
Erythropoietin (%)	44.0	51.0	41.2*	0.003
CAPD/APD (%)	57.0/43.0	63.5/36.5	55.5*/44.5*	0.01/0.02
Conservative treatment (%)	56.2	60.4	54.7	0.093
Serum Albumin (g/dL)(n)	3.6±0.69	3.54±0.78	3.64±0.64	0.295
Hemodialysis previously (%)	44.5	44.4	44.6	0.933
Serum urea (mg/dl)	101.2±24.8	124.5±26.2	101.8±24.9	0.34
Serum creatinine (mg/dl)	8.0±3.1	7.8±3.1	8.1±3.1	0.12
Serum potassium (mEq/L)	4.3±0.6	4.3±0.6	4.4±0.6	0.08
Haemoglobin (g/dl)	11.5±4.0	11.4±3.7	11.5±4.1	0.44

NCA, number of classes of anti-hypertensives in use;

* (E−) *vs* (E+);

SAP: systolic arterial pressure; DAP: diastolic arterial pressure;

MAP: mean arterial pressure.

Analysis of groups divided by the presence of clinically detectible edema

### Subgroup analysis of patients with clinically detectible edema (E+)

**Figure 2:**
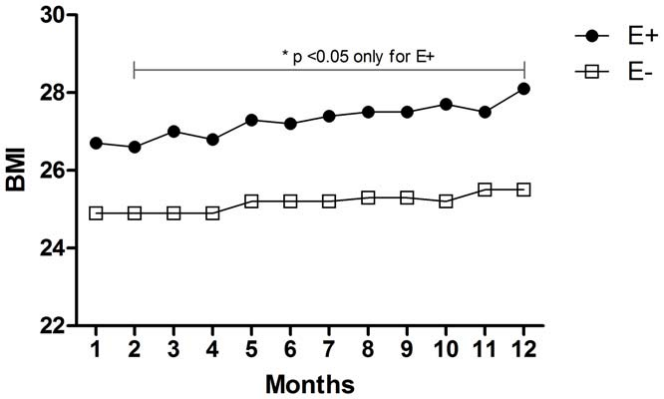
Twelve-month evolution of the body mass index (BMI) in the patient cohort.

During the study, subgroup E+ (n = 307) presented a decrease in SAP between the 1^st^ and 5^th^ month (from 159.5±19.6 to 150.0±25.3 mmHg, *p*<0.05), and SAP remained constant from the 5th month until the end of the study (151.2±30.3 mmHg, *p*>0.05). DAP did not change significantly between the 1^st^ and 12^th^ month (from 90.7±13.3 to 89.0±17.7 mmHg, *p*>0.05). SAP decreased significantly between the 1st and 5th month (from 113.7±13.4 to 108.0±17.2 mmHg, *p*<0.05), and MAP remained constant from the 5^th^ month through the 12^th^ month (109.7±19.8 mmHg, *p*>0.05). NAC did not change between the 1st and 12th months (from 2.3±1.0 to 2.2±1.0 drugs/patient, *p*>0.05). The number of patients with edema decreased between the 2nd and 6th months from 307 to 245 individuals; this number varied through the end of the evaluation period, at which point 243 patients were clinically diagnosed with edema (Figure 1). BMI increased from the 2^nd^ to the 12^th^ month of evaluation (from 26.7±5.1 to 28.1±5.6 kg/m2, *p*<0.05) (Figure 2).

### Subgroup analysis of patients without clinically detectible edema (E−)

**Figure 3:**
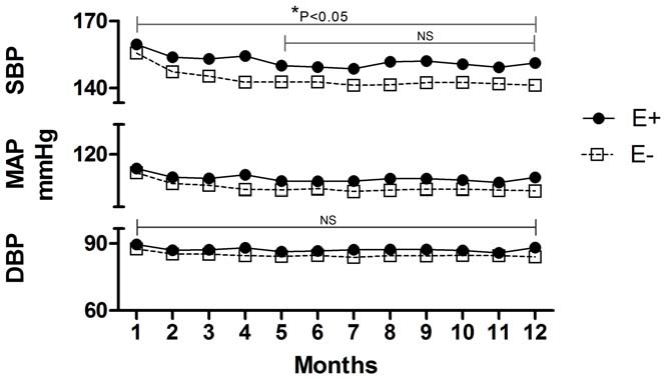
Systolic (SBP), Diastolic (DBP) and Mean Arterial Pressures (MAP) in incident PD patients during 12 months of follow up.

**Figure 4:**
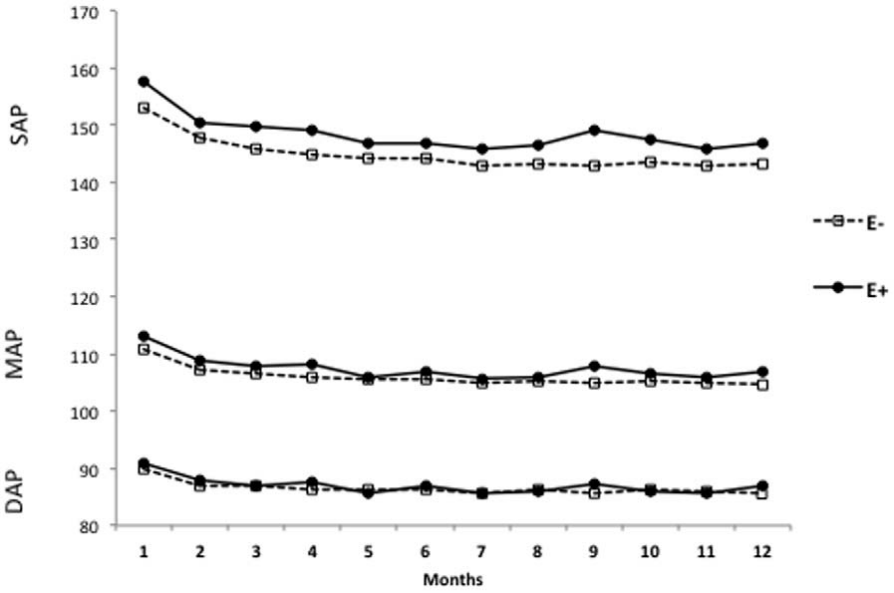
The initial groups (first month) were followed for 12 months.

Subgroup E− (n = 782) presented a significant decrease in SAP between the 1st and 5th month (from 155.6±18.2 to 142.7±24.2 mmHg, p<0.05). After this initial period, SAP remained constant until the end of the study period (141.2±26.6 mmHg, p>0.05). DAP did not change between the 1st and 12th months (89.7±12.5 to 84.7±15.8 mmHg, p>0.05). MAP decreased significantly between the 1st and 5th months (from 111.7±12.6 to 104.1±15.8 mmHg, p<0.05) and then remained constant from the 5^th^ month through the 12^th^ month (103.6±17.9 mmHg, P>0.05). NAC did not vary throughout the study period; the mean at the 1st month was 2.0±0.7, and the mean at the 12th month was 2.1±1.1 (p>0.05). For subgroup E−, there was no difference in BMI during the 12 months of follow-up (Figures 3 and 4)).

### Comparison between the two subgroups of patients

The descriptive characteristics of the two subgroups defined by the presence of edema at the start of dialysis are shown in Table 1. At baseline, subgroup E+ consisted of 307 patients and E− consisted of 782 patients; however, these numbers varied according to monthly clinical evaluations (Figure 1). When only the patients classified E+ and E− in the first month were monitored, the results confirmed the monthly patient classification. E+ and E− move in the same way for the SBP (p = 0.654) although with different mean profiles (p = 0.001). In other words, E+ group showed higher SAP values than E-group during the 12 months period. For the DAP and MAP the trend and mean profile did not show statistical diferences (Figure 4). A comparison of subgroups E+ and E− at the start of treatment (Table 1) revealed significant differences with respect to age (59.6±14.3 vs. 57.7±15.6 years, respectively; p<0.03), BMI (26.7±5.1 vs. 24.9±4.9 kg/m2, respectively; p<0.0001), SAP (159.5±19.6 vs. 155.6±18.2 mmHg, respectively; P<0.001), MAP (113.7±13.4 vs. 111.7±12.6 mmHg, respectively; P<0.01), NAC (2.3±1.0 vs. 2.0±0.7 drugs/patient, respectively; P<0.05) and erythropoietin use (51.0 vs. 41.2%, respectively; P = 0.003). In both subgroups, there were a greater percentage of patients on APD than on CAPD (63.5/36.5 vs. 55.5/44.5%, respectively; p<0.01/0.02). The percentage of patients with diabetes mellitus was greater in subgroup E+ than in subgroup E− (56.0 vs. 37.3%, respectively; P<0.0001), and the number of patients with a history of cardiovascular disease at the start of PD was not significantly different between the two groups (Table 1). SAP, MAP, NAC, and BMI were significantly different between the two subgroups (E+ and E−) in the analysis of the entire follow up period (p<0.05).

## Discussion

It is well known that the expansion of extracellular volume with or without detectible edema is one of the principal factors responsible for the increase in SAP in patients with CKD [3], [9]. In the present study, we observed that SAP and MAP of both subgroups presented a significant decrease in values in the first five months after starting PD therapy and stabilization of these values through the end of the observation period. This behavior was also conferred by Menon *et al.* [17], who reported a reduction in systemic pressures at the start of PD and, contrary to our data, detected an increase in blood pressure levels after 6–12 months on PD. On the other hand, Saldanha *et al.* [7] reported a decrease in blood pressure levels during PD treatment over 5 years, which was associated with the concomitant increase in the number of anti-hypertensive drugs used. In the present study, the initial decline observed in the E+ and E− groups could be attributed to a reduction in extracellular volume as a result of PD [8], [18] because NAC did not change during this period. However, it should be noted that NAC represents a number of anti-hypertensive classes of drugs, which allows for the possibility of variations in the measurement of anti-hypertensive drugs within the same class. On the other hand, NAC maintenance can reflect a non-worsening of SAH in these patients and/or the medical preference to use these drugs for other therapeutic goals such as cardio-protection and/or preservation of residual renal function. Despite the initial decline in arterial blood pressure levels observed in our study, they did not decrease to values within the normal limits; SAP levels were above 140 mmHg during the entire study period. There are other reasons that could explain in the relative control of blood pressure levels in both groups, which are increase activity of the sympathetic nervous system, increase endothelium-derived vasoconstrictors, vascular calcification and activation of the renin-angiotensin system.

**Figure 5:**
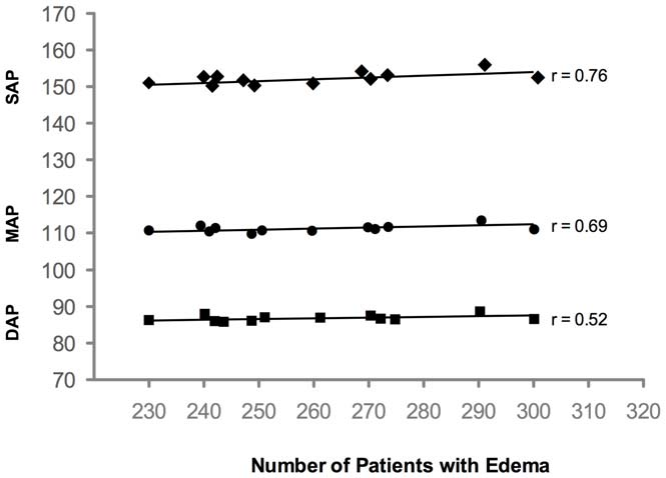
The increase in blood pressure levels correlates positively to the number of patients with edema.

Upon separate analysis of the E+ and E− groups, we observed a monthly variation throughout the study period in the number of patients. This variation was a consequence of bi-directional flow between these groups. Despite this, the number of patients in the E+ subgroup decreased significantly after 12 months, from 307 to 243 patients (Figure 1). Among the E+ subgroup, SAP and MAP levels decreased from baseline until the 5th month, at which time they stabilized until the 12^th^ month (Figure 3 and 4), while DAP did not change significantly during the entire period. In our study, patients with edema exhibited greater blood pressure levels (SAP and MAP) than those observed in the E− subgroup (Figure 3 and 4). Gunal *et al.* [*12*] and Katzarski *et al.* [19] demonstrated that volume overload is an important factor in resistance to SAH treatment for dialysis patients, while Ates *et al.* [*20*] showed that SBP and DBP were negatively correlated with total fluid and sodium removal, as well as with sodium restriction. The increase of blood pressure values was correlated with the number of patients with edema. This association shows that the patients who belonged to the E+ had higher blood pressure levels than those of group E− (Figure 5).

Our data demonstrated that the NAC in the E+ subgroup, despite not varying throughout the study, was significantly greater than in the E− subgroup during the months evaluated. This observation may suggest a greater difficulty in SAH control in the E+ group. Furthermore, BMI in the E+ group increased progressively over the 12 month period. A strong and negative correlation between BMI and the number of patients with edema was observed. This association could be explained in two ways: a worsening of the edema status during PD therapy or a real gain of body mass. We believe that future studies with adequate designs will help to answer this question.

The progressive increase in body weight, likely caused to a large extent by the presence of edema, can be attributed to a water and salt imbalance, the patient's failure to follow medical recommendations, and/or an inadequate PD prescription. The progressive increase in body weight among PD patients might also be attributed to a gain of fat mass due to glucose absorption from the peritoneal cavity, as the patients may have been prescribed more hypertonic PD solutions to improve UF.

In the E− subgroup, blood pressure patterns followed the trend observed in the E+ group and decreased in the first months of PD before subsequently stabilizing (Figure 3). In the E− group, blood pressure levels were lower than those observed in the E+ group during the entire observation period, whereas the NAC in the E− group did not vary significantly during the study period. However, blood pressure values did not reach the normal recommended levels. In general, there are several associated factors that make normalization of blood pressure levels difficult to attain in PD patients, including the presence of diabetes mellitus, aging, and the use of erythropoietin [11], [14], [18]. This was observed in the present study in the E+ group, in which the patients were significantly older and the percentage of patients with diabetes mellitus was significantly greater than in the E− group (Table 1). The significantly larger number of E+ patients who were treated with CAPD as opposed to APD may reflect an inadequate PD prescription, as many of these CAPD patients may be high transporters and/or have UF problems in the long run. Therefore, these patients should have been switched to APD. However, during the observation period, Extraneal was not available in Brazil. Moreover, blood pressures above the normal values could be caused by therapeutic inertia, where soft reasoning often leads to avoidance of intensified therapy by the medical staff [21].

The present study presents several limitations. Edema evaluation cannot be easily standardized, and the influence of expansion or retraction of volume on the systemic pressure levels could be better analyzed if it was evaluated by other methods, such as bio-impedance, inferior vena cava diameter [22], and biomarkers such as ANP [22], [23]. This approach, however, is uncommon in daily medical practice due to the need for tools that are not always available. In addition, the analysis of fluid retention in PD patients is limited by the absence of data regarding residual renal function, the peritoneal membrane solute transport type and UF measurements [9]. Hypoalbuminemia, and consequent water and sodium retention, can explain the presence of edema and the difficulty in normalizing pressure levels; however, an evaluation of the causes of resistance to anti-hypertension therapy was not a focus of this study. It is important to note that the results of this observational study reflect PD practices in Brazil, which may be similar to treatment practices in a large number of countries around the world.

Hypertensive CKD patients experienced a significant reduction in blood pressure levels after the initiation of PD, which was more pronounced in the first few months of therapy. However, most patients do not achieve normalization during the first year of treatment. This difficulty in reducing arterial blood pressure to normal levels is aggravated by the presence of edema, which points to a pivotal role of fluid overload in the hypertension of CKD patients on dialysis. The presence of clinically detectible pitting edema can be a useful clinical sign that could be used to guide the optimization of SAH treatment in patients undergoing continuous peritoneal dialysis.

In summary, volume status is of major importance to outcomes in patients undergoing PD. The lack of a robust edema evaluation and the limited availability of BIA and other objective measures of quantifying volume status make clinicians highly dependent on clinical evaluation. Clinically detectable pitting edema remains the most readily used clinical assessment tool. This study is the first to give a large-scale systematic description of pitting edema in the context of arterial hypertension in PD patients and to assess the effects of edema resolution in blood pressure values with PD initiation.

The results presented here strongly indicate that the presence of such a simple and easily detected clinical sign as pitting edema should be considered to be a relevant observational tool to assess a patient's clinical status, PD prescription and compliance with treatment. The term “back to basics" could mean, “examine your patients, look for edema and observe the blood pressure" and to do this sophisticated technologies are not needed.
